# Recent Progress in the Study of Peroxiredoxin in the Harmful Algal Bloom Species *Chattonella marina*

**DOI:** 10.3390/antiox10020162

**Published:** 2021-01-22

**Authors:** Yohei Shimasaki, Koki Mukai, Yuki Takai, Xuchun Qiu, Yuji Oshima

**Affiliations:** 1 Faculty of Agriculture, Kyushu University, Motooka 744, Nishi-ku, Fukuoka 819-0395, Japan; takai.yuki.665@s.kyushu-u.ac.jp (Y.T.); yoshima@agr.kyushu-u.ac.jp (Y.O.); 2 Department of Biological Sciences, Graduate School of Science and Technology, Kumamoto University, Kurokami 2-39-1, Chuo-ku, Kumamoto 860-8555, Japan; komukai@kumamoto-u.ac.jp; 3 Institute of Environmental Health and Ecological Security, School of Environment and Safety Engineering, Jiangsu University, Zhenjiang, Jiangsu 212013, China; xuchunqiu@ujs.edu.cn

**Keywords:** 2-Cys peroxiredoxin, antioxidant enzyme, growth potential, photosynthesis, oxidative stress

## Abstract

Peroxiredoxin (Prx) is a relatively recently discovered antioxidant enzyme family that scavenges peroxides and is known to be present in organisms from biological taxa ranging from bacteria to multicellular eukaryotes, including photosynthetic organisms. Although there have been many studies of the Prx family in higher plants, green algae, and cyanobacteria, few studies have concerned raphidophytes and dinoflagellates, which are among the eukaryotic algae that cause harmful algal blooms (HABs). In our proteomic study using 2-D electrophoresis, we found a highly expressed 2-Cys peroxiredoxin (2-CysPrx) in the raphidophyte *Chattonella marina* var. *antiqua*, a species that induces mass mortality of aquacultured fish. The abundance of the *C. marina* 2-CysPrx enzyme was highest in the exponential growth phase, during which photosynthetic activity was high, and it then decreased by about a factor of two during the late stationary growth phase. This pattern suggested that 2-CysPrx is a key enzyme involved in the maintenance of high photosynthesis activity. In addition, the fact that the depression of photosynthesis by excessively high irradiance was more severe in the 2-CysPrx low-expression strain (wild type) than in the normal-expression strain (wild type) of *C. marina* suggested that 2-CysPrx played a critical role in protecting the cell from oxidative stress caused by exposure to excessively high irradiance. In the field of HAB research, estimates of growth potential have been desired to predict the population dynamics of HABs for mitigating damage to fisheries. Therefore, omics approaches have recently begun to be applied to elucidate the physiology of the growth of HAB species. In this review, we describe the progress we have made using a molecular physiological approach to identify the roles of 2-CysPrx and other antioxidant enzymes in mitigating environmental stress associated with strong light and high temperatures and resultant oxidative stress. We also describe results of a survey of expressed Prx genes and their growth-phase-dependent behavior in *C. marina* using RNA-seq analysis. Finally, we speculate about the function of these genes and the ecological significance of 2-CysPrx, such as its involvement in circadian rhythms and the toxicity of *C. marina* to fish.

## 1. Introduction

Peroxiredoxin (Prx) is a recently discovered antioxidant enzyme family [1] involved in various signaling pathways as well as antioxidant activity based on peroxide-scavenging ability [2] and is known to be present in organisms found in biological taxa ranging from bacteria to multicellular eukaryotes [3,4,5]. Generally, Prx enzymes can be classified into several groups based on the amino-acid sequence and locations of the cysteine residues. Plants and cyanobacteria have several types of Prx enzymes: 2-CysPrx, 1-CysPrx, PrxQ, and PrxII (or type II Prx), all of which are targeted to different organelles [4]. For example, 2-CysPrx and PrxQ are located in the chloroplast; 1-CysPrx is located in the nucleus and cytoplasm; and PrxIIs are located in the cytosol, mitochondria, and plastids. The Prx enzymes located in the chloroplast of photosynthetic organisms (2-CysPrx and PrxQ) contribute to the removal of hazardous reactive oxygen species (ROS) produced through photosynthetic processes, and they are involved in redox signal control through mechanisms such as retrograde signaling [6]. Many Prx studies have, therefore, involved land plants; cyanobacteria [4]; and eukaryotic, unicellular algae, especially green algae [7]. These studies have gradually revealed the role of Prx enzymes in protecting organisms from various environmental stresses [8] and controlling redox signals [9].

**Figure 1:**
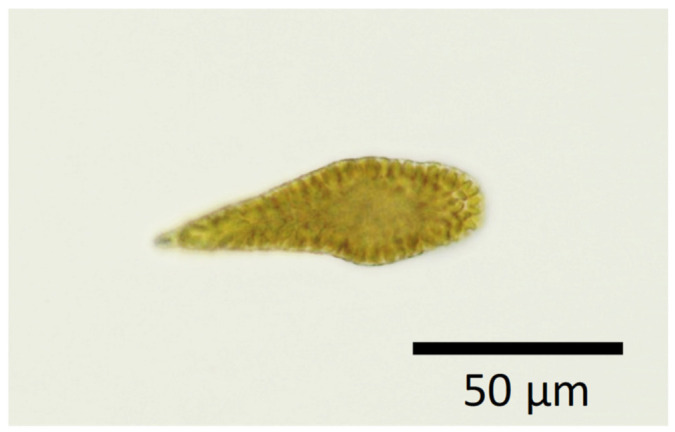
An image of *Chattonella marina* var. *antiqua*.

In contrast, there have been few studies of antioxidant systems, including those that involve Prx, in the eukaryotic algae that cause harmful algal blooms (HABs)—mainly raphidophytes and dinoflagellates—and induce the mass mortality of aquacultured fish. The raphidophyte genus *Chattonella* (Figure 1), in particular, has caused huge economic losses to the aquaculture industry in Japan: approximately JPY 7.1 billion in 1972 in the Harima-nada marine region [10], for example, as well as JPY 2.9 billion in 2009 and JPY 5.3 billion in 2010 in the Yatsushiro Sea [11]. This genus has also caused the mass mortality of cultured southern bluefin tuna in Australia [12] and of salmon in Norway [13]. In the field of HAB studies, it is essential to clarify the environmental factors (e.g., temperature, light intensity, and nutrient concentrations) that affect algal growth and physiology in order to predict bloom dynamics and thereby mitigate damage to fisheries.

Many laboratory [14,15] and field [16,17] studies have investigated the effects of environmental factors on the growth of *Chattonella*. Generally, the formation of phytoplankton blooms is affected by factors such as algal growth, predation pressure, and physical transport processes [18,19,20]. *C. marina* has been reported to grow under a broad range of temperature (15–30 °C) and salinity (10–35) conditions, and its growth rate has been reported to saturate at light intensities of 110 µmol photons m^–2^ s^–1^ [20]. Qiu et al. [21] reported that *C. marina* is capable of growing under severe environmental conditions, including an irradiance as high as ~1000 µmol m^–2^ s^–1^, which is comparable to the average irradiance in surface seawater on a sunny, mid-summer day in coastal areas. However, excessive irradiance is harmful to photosynthetic organisms and may cause high rates of production of ROS. The implication is that the physiology of *C. marina* enables it to resist light-induced oxidative stress during growth and thereby allows it to frequently form dense midsummer blooms in the coastal marine waters of Western Japan. Although Portune et al. [22] investigated antioxidant activity of harmful raphidophytes, few molecular biological studies related to antioxidant activities in HAB species, including *C. marina*, have been undertaken until recently.

A link between antioxidant enzymes and stress tolerance has been recognized for a broad range of organisms, including photosynthetic species. Miao et al. [23] reported that a mutant of the thale cress, *Arabidopsis thaliana*, contains a glutathione peroxidase (GPX) that enables it to tolerate a higher rate of water loss when stressed under drought conditions; this strain exhibits higher sensitivity to hydrogen peroxide (H_2_O_2_) treatment during seed germination and seedling development and produces more H_2_O_2_ in guard cells than wild-type strains. Insertion into the yeast of genes from the microalga *Chlorella vulgaris* Beijerinck that encode the antioxidant enzyme peroxiredoxin and NADPH-dependent thioredoxin reductase increases the tolerance of the yeast to freezing, heat, and oxidative stresses [24]. Knock-down strains of rice that contain functional ascorbate peroxidase (APX) isoforms exhibit earlier senescence than wild-type strains [25]. Antioxidant enzymes may thus be closely related to abiotic stress tolerance and growth potential.

*C. marina* generates higher amounts of ROS, such as the superoxide anion (O_2_^−^) and H_2_O_2_, than other unicellular algae [22,26]. Growth activity in this species is also inhibited by decomposition of O_2_^−^ and H_2_O_2_ caused by addition of superoxide dismutase (SOD) and catalase (CAT) [27]. A certain level of oxidative stress by O_2_^−^ and H_2_O_2_ might, therefore, have a beneficial effect on *C. marina* growth. However, because ROS attack various cellular molecules such as DNA, RNA, proteins, and lipids [28,29], high concentrations of ROS can have deleterious effects on growth. In mammals and plants, lipid peroxidation is caused by oxidative stress from ROS and non-radical reactions mediated by lipoxygenase [30,31,32]. Therefore, *C. marina*, which generates relatively high amounts of ROS compared to other unicellular algae, is probably exposed to a high degree of oxidative stress, and it might protect itself from ROS with a sophisticated system involving the use of antioxidants.

In many cases, HAB species form dense blooms during the summer under harsh environmental conditions characterized by supra-optimal irradiance and temperature. Understanding the physiological mechanisms that allow algae to tolerate these environmental conditions is important to biological and fisheries science. Recently, omics analysis has begun to be used to investigate the comprehensive molecular mechanisms that account for HAB phenomena [21,33,34,35,36,37,38]. Our studies have focused on the molecular mechanisms that control the growth of HAB species. Through our proteomic study, we identified proteins with a growth rate-dependent expression pattern, and we found that 2-CysPrx [21] was a possible key factor that enabled the HAB species *C. marina* to maintain a high cell division rate. In this review, we describe the progress we have made via a series of functional analyses of 2-CysPrx and other antioxidant enzymes in elucidating how *C. marina* acclimates to environmental stresses such as strong light, high temperature, and resultant oxidative stress. We also present the results of current studies of the expression of the Prx family of enzymes and the growth phase-dependent response of their gene expression in *C. marina* using RNA-seq analysis. Finally, we include some speculations about the function of Prx enzymes during algal growth and involvement of the 2-CysPrx enzyme in circadian periodicity and the toxicity of *C. marina* to fish.

## 2. Temporal Changes of Levels of 2-CysPRX Enzymes During Algal Growth

**Figure 2:**
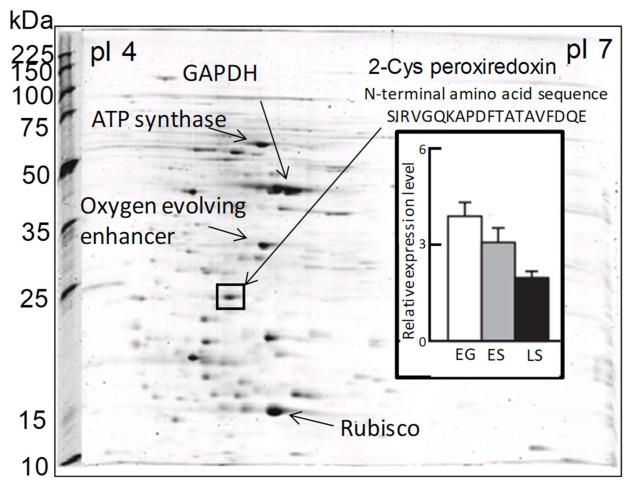
Two-dimensional gel electrophoresis profiles of *Chattonella marina* var. *antiqua* and some identified major protein spots, including 2-CysPrx. The bar graph shows the levels of 2-CysPrx protein expression in the exponential growth phase (EG), early stationary phase (ES), and late stationary phase (LS) [21].

In a previous proteomic study using two-dimensional electrophoresis [21], we detected a highly expressed protein in *C. marina* var. *antiqua* (NIES-1 strain) that was identified as 2-CysPrx based on the 20 residues of its N-terminal sequence (Figure 2). The abundance of the *C. marina* 2-CysPrx enzyme was highest during exponential growth when photosynthetic activity was high; the abundance then decreased roughly twofold during the late stationary phase (Figure 2). This protein accounted for 4% of all the proteins detected in an exponential-growth-phase sample of *C. marina* cells based on the fluorescence intensity of all protein spots in the 2-DE profile. If consideration is given to the difference between measurement methods, our estimation of the protein abundance was nearly equal to or higher than estimates reported in previous studies, which have indicated that the contributions of Prx (0.5–1%) to cellular protein are relatively high in several types of cultured cells [39]. The 2-CysPrx in *C. marina* is, therefore, highly expressed at the protein level and is thought to facilitate cell proliferation by removing peroxides produced during photosynthesis.

**Figure 3:**
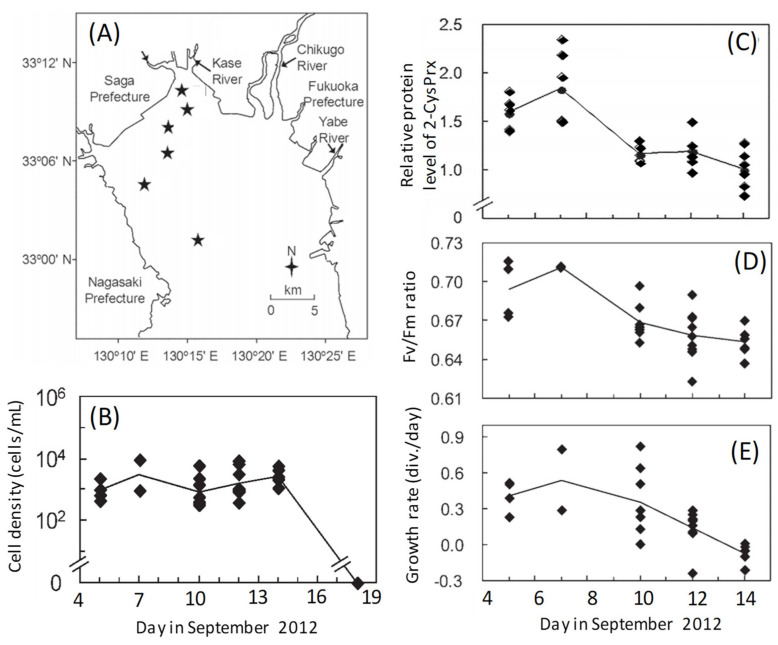
Field sites for sampling from 5 to 18 September 2012 in the inner part of the Ariake Sea, Japan (**A**). Stars indicate the sampling stations. Temporal variations of the (**B**) cell concentrations, (**C**) relative 2-CysPrx expression levels, (**D**) photosynthetic activities (Fv/Fm ratios), and (**E**) growth of *Chattonella marina* cells in natural seawater samples. Growth rates were calculated from the change of cell concentrations between the sampling day and the next day after incubation in a growth chamber. Symbols indicate values for each station, and lines indicate the average values of all stations on the same sampling day [40,41].

A growth phase-dependent change of the level of expression of the 2-CysPrx protein and photosynthetic activity has also been observed during a natural bloom [40,41]. We investigated temporal variations of protein expression profiles in *C. marina* cells during a HAB (5–14 September 2012) that occurred in the inner part of the Ariake Sea to the north of the island of Kyushu, Japan (Figure 3A,B). Proteomic analyses revealed a decreasing trend in the abundances of 2-CysPRX (Figure 3C) and other photosynthesis-related proteins such as LHCP 4, Cyt c553, and GAPDH [41] as the bloom progressed. The maximum potential quantum yield of photosystem II (Fv/Fm ratio) decreased in a manner similar to the daily growth rate, which was calculated from the change of cell concentrations in a growth chamber between the sampling day and the next day (Figure 3D,E). The abundances of the above proteins were significantly (*p* < 0.05) and positively correlated with Fv/Fm ratios and daily growth rates [41].

The antioxidant function of 2-CysPrx in photosynthetic organisms has been investigated in higher plants [42,43,44,45] and cyanobacteria [3,46,47]. Expression of the 2-CysPrx genes is under developmental control, and steady-state mRNA levels decrease with tissue age in barley [43], *Arabidopsis thaliana* [44], and *Riccia fluitans* [45]. In cyanobacteria, the Fv/Fm ratio and growth rate under high irradiance are lower in a mutant strain of *Synechocystis* 6803 lacking 2-CysPrx than in wild-type cells [46]. Our discovery that the abundance of 2-CysPrx was positively correlated with both Fv/Fm ratios and growth rates was consistent with the results of those earlier studies. *Chattonella* is known to produce high levels of ROS as a byproduct of photosynthesis [22,48] or possibly of another pathway [49], and they, therefore, require large amounts of antioxidant enzymes to prevent damage from ROS during active growth. As a *Chattonella* bloom progresses, relatively low amounts of 2-CysPrx may provide insufficient protection, and the resultant accumulation of peroxides within cells can impair photosynthesis by damaging chloroplast structures [44] and cause a rapid termination of the bloom (Figure 3B) by triggering peroxide signaling events that lead to cell death [50]. Thus, further discussions are needed concerning the growth phase-dependent physiological function of 2-CysPrx content.

## 3. Structure of the 2-CysPrx Gene in *Chattonella marina*

To elucidate the mechanism responsible for the production of 2-CysPrx, we determined the structure of the 2-CysPrx gene and the predicted amino acid sequence using the rapid amplification of cDNA ends (RACE) method [51]. The open reading frame of the 2-CysPrx gene was 585 base pairs (bp) long and encoded a protein consisting of 195 amino acids. The putative amino acid sequence contained two cysteine residues located at the 49th and 170th amino acid positions from the N-terminal methionine residue. The sequence also possessed 2-CysPrx characteristic motifs, F (FFYPLDFTFVCPTEI), GGLG, EVCP, and YF [50]. We concluded that the 2-CysPrx gene is located in the chloroplast genome, because there was no intron in the DNA sequence, and the position of the 2-CysPrx gene relative to several other genes (*ycf59*–*2-CysPrx*–*rpl35*–*rpl20*) was the same as the position of the corresponding gene in the chloroplast genome of the raphidophyte *Heterosigma akashiwo* (GenBank accession no. EU168191.1) [52]. In addition, inverse polymerase chain reaction (PCR) analysis revealed possible TATA and GGA motifs upstream of the 2-CysPrx gene that were recognized by nuclear-encoded plastid RNA polymerase (NEP) as well as a possible −10 and −35 box recognized by plastid-encoded plastid RNA polymerase (PEP). These results suggested the possibility that the *C. marina* 2-CysPrx gene is found in the chloroplast, and its transcription can be regulated by both NEP and PEP [51].

A previous study [53] reported that the chloroplast-encoding gene is transcribed by NEP or PEP. In general, PEP involves photosynthesis-related genes, and NEP involves housekeeping genes. The authors mentioned three types of NEP (Ia, Ib, and II). Type Ib NEP recognized the sequence YRTA (TATA) motif near the transcription–initiation site and a conserved GGA motif about 18–20 bp upstream of the YRTA motif. The YRTA motif was also reported by Joshi [54] to be located 32 ± 7 bp upstream of the transcription–initiation site. In the case of *C. marina*, the TATA motif was found 27 bp upstream of the transcription–initiation site, and the GGA motif was found 18 bp upstream of the TATA motif. In contrast, Börner et al. [53] noted that PEP recognizes bacterial σ70 promoters of the −10 (TATAAT) and −35 box (TTGACA). We observed the sequences TAAAAT around 10 bp and TTGATC around 35 bp upstream of the transcription–initiation site in the *C. marina* 2-CysPrx gene. Those sequences resembled the −10 and −35 box in the bacterial σ70 promoters. The *C. marina* 2-CysPrx gene thus had similar characteristics of both the NEP and PEP promoters in its transcription–regulation site. Magee and Kavanagh [55] reported that transcriptional patterns in plastid genes and operons can be assigned to three classes, those that contain (1) PEP promoters only, (2) both PEP and NEP promoters, and (3) NEP promoters only. There is hence a possibility that the *C. marina* 2-CysPrx gene is transcribed by NEP and PEP promoters, although the NEP gene (*rpoT;3*) remains undiscovered in marine algae [56]. Further analysis will be needed to reveal the mechanisms that regulate the 2-CysPrx gene in *C. marina*.

## 4. Induction of Gene Expression for the Production of Antioxidant Enzymes by Light, Thermal, and Oxidative Stress under Laboratory Conditions

**Figure 4:**
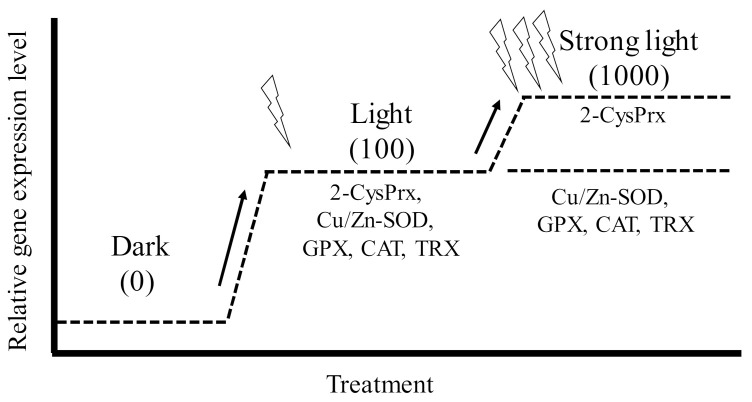
Conceptual diagram of gene expression pattern of six antioxidant enzymes (2-CysPRX, Cu Zn superoxide dismutase (Cu/Zn-SOD), glutathione peroxidase (GPX), catalase (CAT), and thioredoxin (TRX)) in *Chattonella marina* under conditions of elevated irradiance. The numbers in parentheses are light intensities (μmol photons m^−2^ s^−1^) [57].

**Figure 5:**
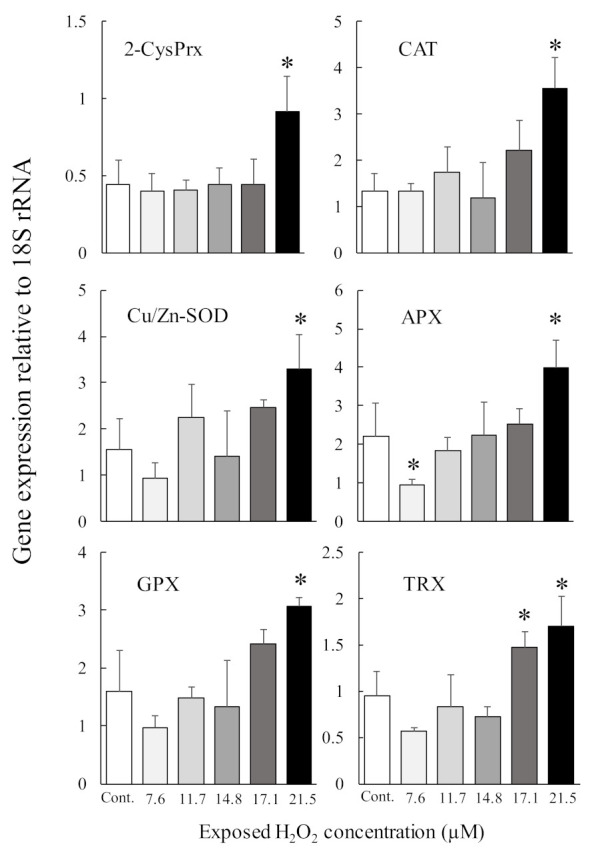
Levels of gene expression of six antioxidant enzymes (2-CysPRX, Cu Zn superoxide dismutase (Cu/Zn-SOD), glutathione peroxidase (GPX), catalase (CAT), ascorbate peroxidase (APX), and thioredoxin (TRX)) determined by quantitative PCR in *Chattonella marina* after one day of exposure to various concentrations of H_2_O_2_. The H_2_O_2_ concentrations on the ordinate of the figure were measured by the method described in Mukai et al. [51]. Values are means ± SD of four replicates. Asterisks indicate significant differences in expression levels compared with control (*p* < 0.05 by Dunnett’s test).

Antioxidant enzymes are essential proteins that maintain algal cell proliferation potential by protecting against oxidative stress induced by photosynthetic processes. We, therefore, investigated the induction of the genes that code for the production of 2-CysPrx and five other antioxidant enzymes (Cu Zn superoxide dismutase (Cu/Zn-SOD), glutathione peroxidase (GPX), catalase (CAT), ascorbate peroxidase (APX), and thioredoxin (TRX)) under different light, oxidative stress, and temperature (2-CysPrx only) conditions using quantitative PCR [51,57,58]. In dark-acclimated cells, expression levels of all antioxidant enzymes except APX were significantly increased by light irradiation (100 μmol photons m^−2^ s^−1^), and these expression levels decreased within 24 h, whereas the expression of the 2-CysPrx gene remained high for 24 h [57]. However, in *C. marina* cells acclimated to 100 μmol photons m^−2^ s ^−1^, only the levels of expression of the 2-CysPrx gene were significantly (*p* < 0.05) increased by strong light irradiation (1000 μmol photons m^−2^ s^−1^) (Figure 4). In that experiment, both the H_2_O_2_ concentration and scavenging activity also increased [51]. Extracellular exposure to H_2_O_2_ at 21.5 μM (Figure 5, manuscript in preparation) induced expression of the genes that code for the production of six antioxidant enzymes, and 100 μM of H_2_O_2_ [59] also induced expression of the 2-CysPrx gene. In addition, we observed thermal inductivity of the 2-CysPrx gene [58]. The expression of the *C. marina* 2-CysPrx gene at 30 °C was 6.1 (day 1) and 10.3 times (day 2) the expression at 10 °C and 1.5 (day 1) and 5.2 times (day 2) the expression at 20 °C. Our previous study [60] showed that photosynthetic activity increases with increasing temperature in *C. marina.* In general, high photosynthetic activity increases intracellular oxidative stress. Genes that code for the production of antioxidant enzymes are thought to facilitate the removal of intracellular oxidative stress, and the 2-CysPrx enzyme in *C. marina* may help to alleviate intracellular oxidative stress so that photosynthetic activity and growth can be maintained under environmental conditions characterized by strong light and high water temperatures.

However, the light inductivity of the expression of these genes, including the 2-CysPrx gene, was completely suppressed by treatment with the photosystem II inhibitor diuron at 10 µM within 1 or 3 h after exposure (manuscript in preparation). The redox state of the photosynthetic electron transport chain, trans-thylakoid potential, and ROS are known to be triggers of plastid-to-nucleus retrograde signaling [6]. Five antioxidant genes, but not the 2-CysPrx gene, are thought to be nuclear-genome-encoded antioxidant enzymes [61,62,63,64,65]. These five genes should, therefore, be regulated by plastid-to-nucleus retrograde signaling pathways, and the difference in the source genomes between 2-CysPrx and the other five antioxidant enzymes may be involved in the difference of the observed expression patterns under strong-light conditions (Figure 4).

## 5. Effect of 2-CysPrx Protein Levels on Survival and Production of Lipid Peroxide under High Irradiance

**Figure 6:**
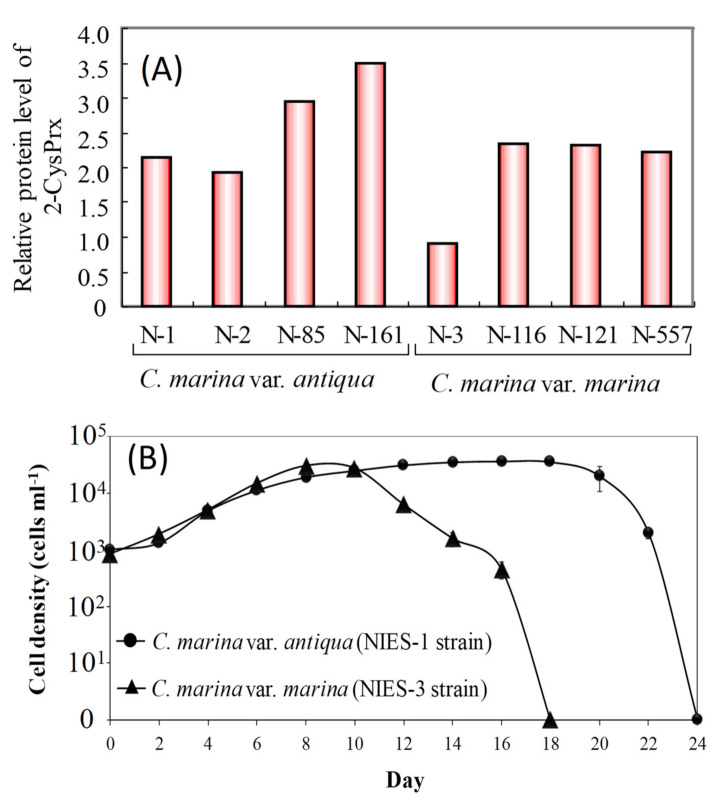
Difference of 2-CysPrx protein expression levels in *Chattonella marina* strains (**A**) and cell density versus culture duration in NIES-1 (2-CysPrx-normal-expression strain) and NIES-3 strains (2-CysPrx-low-expression strain) (**B**).

Previous studies have investigated the relationships between cell proliferation and the accumulation of oxidative damage in photosynthetic organisms and have revealed that an increase in intracellular lipid peroxide (LPO) decreases survival rates and potentials for growth during certain phases of growth and under various stressful conditions including postflooding, exposure to herbicides, and exposure to ultraviolet radiation [66,67,68,69]. Hurng and Kao [67] reported an increase in LPO activity and decrease in SOD and CAT activities in tobacco leaves after flooding. In addition, ascorbate acid (vitamin C) concentrations increase, and the activities of glutathione and glutathione reductase increase as a primary response to increased LPO in beans that are exposed to the herbicide acifluorfen-sodium [66]. LPO, a frequently used indicator of oxidative damage, should, therefore, affect photosynthetic activity and cell proliferation in phytoplankton, including species of *Chattonella*. In contrast, in our proteomic study using 2-D electrophoresis (unpublished data), we found that the amount of the antioxidant enzyme 2-CysPrx protein in a *C. marina* NIES-3 strain was less than half of that in seven other cultured *Chattonella* strains (Figure 6A), and the time from initiation of culture (1000 cells mL^−1^) to the decline in cell numbers for the NIES-3 strain was less than that of the NIES-1 strain (Figure 6B) grown at 25 °C under 230 μmol m^−2^ s^−1^ and a 12 h light:12 h dark cycle. Because peroxidase degrades H_2_O_2_, strains such as NIES-3 that express low amounts of 2-CysPrx may be less capable of protecting themselves from H_2_O_2_ oxidative stress and show reduced maintenance of photosynthetic and growth activities.

We, therefore, investigated the differences in the short-term responses of growth and intracellular LPO concentrations between *C. marina* NIES-1 (2-CysPrx-normal-expression strain) and NIES-3 (2-CysPrx-low-expression strain) grown at high irradiances. Before this test, both strains were cultured until the early stationary phase of growth (NIES-1: 11,000 cells mL^–1^, NIES-3: 19,000 cells mL^–1^) at 25 °C under a 14 h light:10 h dark photoperiod with a photoperiod irradiance of 110 µmol photons m^–2^ s^–1^. Sixty milliliter cell suspensions in plastic flasks were then cultured at 20, 110, or 520 µmol photons m^–2^ s^–1^ of continuous irradiance by LED bulbs (low, moderate, or high irradiance, respectively) for 13 h (N = 4). During exposure, an 8.5 mL cell suspension was sampled from each flask at 1, 7, and 13 h. Soon after sampling, a portion of each cell suspension was assayed for cell counts, measurement of concentrations of O_2_^−^ [70] and H_2_O_2_, and evaluation of photosynthetic activity [51]. The remaining cells were pelletized by centrifugation at 1800 × g for 10 min, and the cell pellet was stored at −80 °C until LPO analysis (Lipid Hydroperoxide Assay Kit, Cayman Chemical, Ann Arbor, MI, USA).

**Figure 7:**
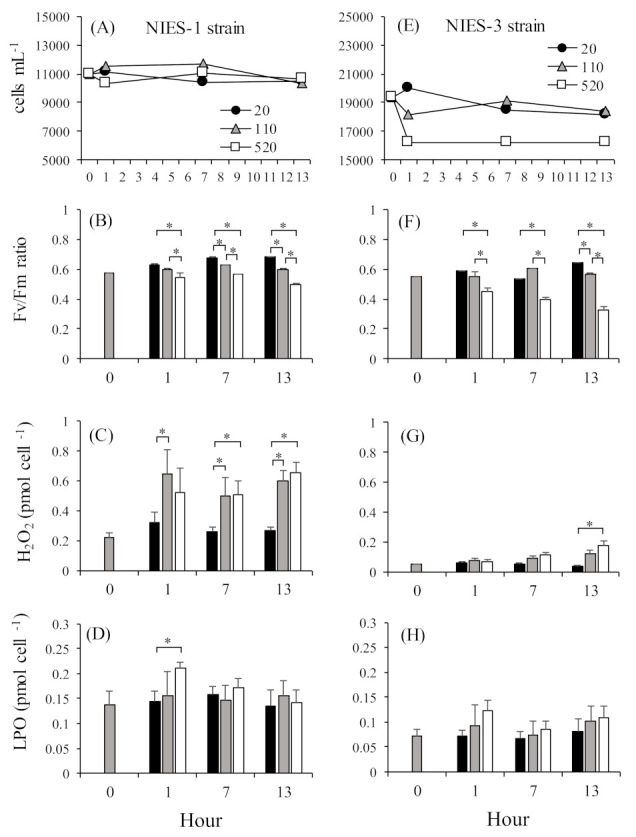
Change of cell densities, Fv/Fm ratios, H_2_O_2_ concentrations, and lipid peroxide (LPO) concentrations in NIES-1 (**A**–**D**) and NIES-3 (**E**–**H**) strains during 13 h of exposure to different light intensities (20, 110, and 520 μmol photons m^−2^ s^−1^). Values are means ± SDs (N = 4). Asterisks indicate significant differences in expression levels (*p* < 0.05 by Tukey–Kramer and Scheffe’s F test).

Cell densities in the NIES-1 strain were relatively stable at all irradiances for 13 h (Figure 7A). In the NIES-3 strain, cell densities in the low- and moderate-light treatments were relatively stable throughout the experimental period, but the average cell density in the high-light treatment declined just after exposure and then stabilized for the remainder of the experiment (Figure 7E). In both the NIES-1 and NIES-3 strains, almost all the Fv/Fm ratios in the high-irradiance treatments were significantly lower than those in the low- and moderate-irradiance treatments (Figure 7B,F). The mean percent inhibition in the high-irradiance treatment was greater in the NIES-3 strain (38.8 ± 10.1%) than in the NIES-1 strain (20.2 ± 4.8%). Irradiances at the compensation point (Io) and growth saturation (Is) in *C. marina* were 10.31–10.51 μmol m^−2^ s^−1^ and 110 μmol m^−2^ s^−1^, respectively [20]. Thus, *C. marina* cells exposed to moderate (110 μmol m^−2^ s^−1^) and high irradiance (520 μmol m^−2^ s^−1^) presumably received an excess of light energy, which would have led to photoinhibition. In addition, a significant irradiance-dependent increase in H_2_O_2_ and LPO concentrations were observed for both the NIES-1 and NIES-3 strains (Table 1, Figure 7C,D,G,H).

Production of O_2_^−^ and H_2_O_2_ can be induced by excessive electron transport in photosystem II [71,72,73]. A key protein in PSII, D1, is decomposed by O_2_^−^, and the singlet oxygen generated by the over-reduction in states within photosystem II when it is exposed to high light leads to decreased photosynthetic activity [74,75]. This mechanism could explain the decrease in photosynthetic activity associated with excessive light energy. However, no significant increase in O_2_^−^ concentrations was observed in our data (Table 1). During the photosynthetic process, O_2_^−^ is produced by one-electron reduction of oxygen (O_2_) at the reduction side of PSI in the Mehler reaction. This phenomenon has been thought to occur in *C. marina*, but Shimada et al. [76] reported that *C. marina* produces large amounts of O_2_^−^ at its cell surface through non-photosynthetic processes that may involve NADPH oxidase. It may, therefore, not have been possible to detect an increase in photosynthesis-related O_2_^−^ production under conditions of high irradiance.

In mammals and plants, lipid peroxidation is caused by oxidative stress from ROS and non-radical reactions involving, for example, lipoxygenase [30,31,32]. The hydroperoxyl radical (LOO•) is generated by reactions with oxygen molecules and lipid-derived free radicals (L•) generated as a product of reactions involving hydrogen atoms in lipid molecules (LH) and •OH [29]. LPOs produced by reactions between LOO• and hydrogen atoms in other LHs may generate additional L• radicals. The •OH radical is thus a trigger for LPO formation; •OH is generated via the reaction of H_2_O_2_ and a transitional metal ion (such as the iron cation) in the Fenton reaction during the process of ROS production [77]. The Fenton reaction also occurs by the reaction between H_2_O_2_ and reduced iron-sulfur in PSI. LPO is generated by ^1^O_2_ produced by the photosensitizing reaction. The large amounts of ROS, such as H_2_O_2_, •OH, and ^1^O_2_, produced under excessive irradiance should increase the risk of LPO production. Accumulation of LPO increases with increased oxidative stress [66,67,68,69]. Because the H_2_O_2_ concentration was higher in the high-irradiance treatment than in the low-irradiance treatment, the increased LPO in *C. mairna* might have been caused by excessive irradiance.

As described previously, the amount of 2-CysPrx in the NIES-3 strain was less than half of that in other *C. marina* strains, including NIES-1. The *C. marina* NIES-1 and NIES-3 strains manifested different physiological responses, especially in terms of decreased levels of Fv/Fm and cell densities. The mechanisms underlying these differences are unclear, but a mutant strain of *Synechocystis* 6803 lacking 2-CysPrx and grown under high irradiance has exhibited a lower Fv/Fm ratio and growth rate than wild-type cells [46]. The fact that the quantities of the 2-CysPrx protein were lower in the NIES-3 strain than in the NIES-1 strain may, therefore, have involved reduced resistance to excessive light energy.

We determined the physiological response to oxidative stress caused by high irradiance, LPO formation, and decreased cell viability in the *C. marina* NIES-1 and NIES-3 strains. However, low-molecular-weight antioxidants (such as polyphenol and tocopherol [vitamin E]) suppress LPO accumulation [78,79]. Tocopherol can terminate chain reactions of free radicals of polyunsaturated fatty acids generated by lipid oxidation [80]. In addition, production of tocopherol and polyphenol is induced by high irradiance in higher plants [81]. Further study, including investigations of the antioxidant function of low-molecular-weight substances, is thus needed to understand the anti-oxidation system in representative HAB species such as *C. marina*.

## 6. Survey of Prx Family of Genes Expressed in *Chattonella marina* by RNA-seq Analysis and Possible Application for Evaluation of Cell Division Activity

Comprehensive analysis of growth-phase-dependent gene expression will provide useful information about the molecular mechanisms that maintain growth potential and the molecular indicators that are candidates for monitoring growth potential in *C. marina*. Recently, RNA-seq analysis has been performed on harmful algae [34,35,36,38]. In our current study, RNA-seq analysis is being performed using *C. marina* cells in the exponential phase as well as the early-, mid-, and late-stationary phases of growth (manuscript in preparation). RNA sequences were read by paired-end sequencing (maximum lead length 2 × 150 bp) using an Illumina NovaSeq 6000 system. The tentative RNA-seq analysis resulted in the acquisition of approximately 10.7–13.8 million paired-end reads in each sample from the total RNA of *C. marina*. After the trinity process, 186,997 contigs of mean length 790 bp were generated, and after the TransDecoder process, 139,676 contigs were converted to amino acid sequences.

**Figure 8:**
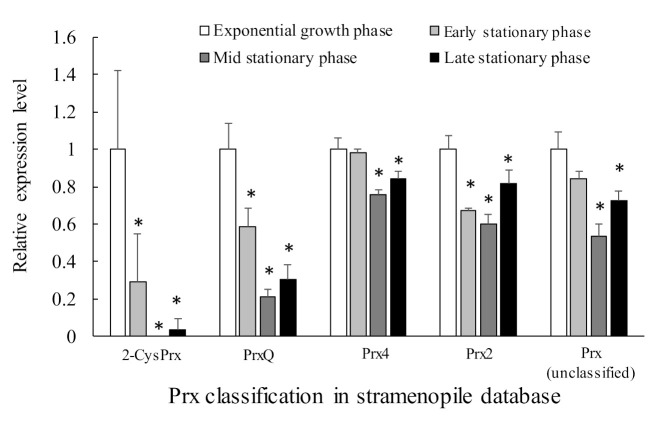
Relative gene expression levels based on the expression level of each gene during the exponential growth phase by RNA-seq analysis. Table 2 provides detailed descriptions of the Prx genes. Values are means ± SD of three replicates. Asterisks indicate significant differences in expression levels compared with that in the exponential growth phase (*p* < 0.05 by Dunnett’s test).

About 11 sequences of genes possibly belonging to the Prx family were identified. Among them, five were typical 2-CysPrx, four were atypical 2-CysPrx, and two were 1-CysPrx. Table 2 lists the five genes that were expressed relatively highly, and Figure 8 shows their relative expression levels in each growth phase. The expression levels of these five genes tended to be highest during exponential growth. This pattern was especially true for the 2-CysPrx and PrxQ genes, which are localized in the chloroplast and are thought to play a role in removing the ROS byproducts of active photosynthesis during exponential growth. Their expression declined remarkably during the stationary growth phases. This pattern was very consistent with the role of 2-CysPrx and PrxQ in the removal of ROS and their involvement in redox control within the chloroplast. In addition, the clearly growth-phase-dependent changes of their gene expression mean that these genes are promising candidate biomarkers for predicting the dynamics of *C. marina* blooms in the field. On the other hand, gene expression levels of Cu/Zn-SOD, GPX, CAT, APX, and TRX were almost stable and did not show growth phase-dependent fluctuation (manuscript in preparation). 

Other photosynthetic-related proteins also showed growth-phase-dependent fluctuation in gene expression. In this RNA-seq analysis, there was a significant decrease over time of 1036 genes related to synthesis of ATP and of proteins that are components of photosystems and that were identified by a BlastP search of the NCBI database (stramenopiles). Photosynthetic organisms can produce ATP by converting light to chemical energy. Light energy captured by a light-harvesting complex flows through an electron-transport chain within the chloroplast. A proton gradient across the thylakoid membrane and stroma then causes an input of protons via plastoquinone and the cytochrome *b*_6_*f* complex [83,84], and ATP synthase uses the proton gradient to produce ATP [85]. Sufficient chemical energy could, therefore, not have been produced by the smaller quantity of proteins that were components of photosystems and ATP synthesis-related proteins during the late phase of growth. The Fv/Fm ratio gradually decreased during the *C. marina* culture period, and it became extremely low during the decline phase [21]. The decline in this population may have been closely related to a reduction in photosynthetic and ATP-synthesis activity as well as antioxidant activity.

## 7. Ecological Significance of Circadian Rhythms in HAB Species

Natural populations of some flagellates are well known to display diurnal vertical migration behavior, in which individuals swim upward during the day (to acquire light for photosynthesis) and downward at night (to take up nutrients within the nutricline) throughout a diurnal cycle [86,87,88]. Because *Chattonella* cells tend to accumulate in the surface water during midday in summer [89], a strong tolerance to excessive light exposure is necessary to avoid photooxidative damage. During the process of downward migration, the rapid recovery of photosynthetic activity may help *Chattonella* cells to store more energy and produce more organic matter needed for vital functions. These photoprotective mechanisms may provide an explanation for the fact that *Chattonella* species can maximize photosynthesis and grow well under high-irradiance conditions [21,88,90]. During diurnal vertical migration, various biological characteristics of *Chattonella* species, especially those related to photosynthesis, have also been reported to display clear diurnal changes. Those characteristics include phosphate metabolism [91], nitrate reductase gene expression [92], cell division [93], and production of reactive oxygen species (ROS) [94]. The regulation of the diurnal variation of these biological characteristics of *Chattonella* species is complex. The process of regulation may involve an endogenous circadian rhythm [88,95], light–dark cycling in coordination with photosynthetic activity [94,96], or both. 

The antioxidant function is also important for protecting cells from oxidative stress under excessive light. An efficient circadian rhythm could, therefore, facilitate maintenance of active cell division. Among antioxidant enzymes, the 2-CysPrx protein is well known to be a circadian rhythm marker in a broad range of organisms [97]. Circadian rhythms are also important for the efficient use of sunlight energy in photosynthesis by eukaryotic, unicellular algae. In our current study, a circadian rhythm was observed in the mRNA levels of 2-CysPrx encoded in the chloroplast genome of *C. marina* (manuscript in preparation). In an experiment under a 12 h light:12 h dark photoperiodicity, the level of expression of the 2-CysPrx gene gradually increased during the dark period, rapidly increased just after the beginning of the photoperiod, and then gradually decreased toward the end of the photoperiod. This rhythm continued after the cells were transferred to 24 h of darkness. In contrast, proteomic analysis revealed no clear circadian rhythm in the level of the 2-CysPrx protein [98]. Translation control as well as transcriptional control may, therefore, be important for the chloroplast-encoded 2-CysPrx gene of *C. marina*. We have now started to study the mechanism of both translational and transcriptional regulation of the Prx protein family. Our study of Prx in HAB species from the standpoint of evaluating growth potential has only just begun, and gene-editing technology is not well-established in HAB species. Further study will be needed to clarify the molecular mechanisms that enable maintenance of high rates of photosynthesis and growth under light and thermal stress.

## 8. Possibility of Relationship between Antioxidant Activity and Toxicity to Fish

In coastal areas of Japan, HABs of *Chattonella* species have repeatedly caused massive mortalities of cultured fishes, especially yellowtail *Seriola quinqueradiata* [99]. Many exposure tests and field investigations have been conducted to understand the effects of *Chattonella* blooms on fish. These tests have examined mortality, tissue damage, and some physiological responses in the gills and blood as endpoints [100,101,102,103]. These studies have provided valuable information for obtaining baseline toxicity data used for the development of strategies to reduce the damage from HABs. Because suffocation induced by *Chattonella* is the final cause of fish death, some countermeasures that can decrease fish movements and their requirements for oxygen have been developed, and these have been shown to be effective in mitigating the negative impacts of *Chattonella* HABs on fishes, particularly cage-cultured yellowtail [99].

Some studies [104,105] have suggested that *C. marina* kills fishes by damaging fish gills, and the ROS, which are produced on the surfaces of *C. marina* cells [106,107], are suspected to be the agents responsible for injuring the gill tissues. However, the mechanism by which *Chattonella* spp. kill fish remains controversial, because multiple factors are involved in the ichthyotoxicity of *Chattonella* spp. [101,102]. Although the concentrations of ROS released by *Chattonella* spp. seem to be insufficient to kill fish directly [101,102], it has been demonstrated that ROS and other toxins play synergistic roles in ichthyotoxicity [108], and activities that lead to the production of ROS are an important factor that influences ichthyotoxicity [109,110].

Because previous studies have revealed that the extent of O_2_^−^ production differs among *C. marina* strains [109,111], the mechanism responsible for high rates of ROS production in *Chattonella* species has been investigated. Shimada et al. [76] reported that this species produces large amounts of O_2_^−^ at the cell surface through non-photosynthetic processes that may involve NADPH oxidase. Yuasa et al. [106] carried out an experiment that involved the use of inhibitors of photosynthetic electron transport and the Calvin–Benson cycle; on the basis of their results, they suggested that reducing power derived from electron transport in photosystem II is required for the production of O_2_^−^ and that the accumulation of NADPH stimulates its production. However, they also reported the possibility of a non-photosynthetic pathway that produced O_2_^−^ extracellularly [49]. The mechanism responsible for the toxicity of *C. marina* to fish is, therefore, thought to be complicated and is now under analysis. In our RNA-seq analysis, we identified the Contig “6598c0g1i1” (Table 2) as a Prx4 gene in the stramenopile database. The Prx4 is a secreted protein in mammals. If this Prx protein is secreted to the *C. marina* cell surface, the secreted Prx (or a Prx membrane protein) may affect the toxicity of *C. marina* toward fish by decreasing ROS levels on the cell surface. Immunochemical techniques against cell surface protein may be used to test this hypothesis in the future.

## 9. Conclusions

The discovery of the antioxidant enzyme peroxiredoxin was relatively recent. Peroxiredoxin has peroxide-scavenging ability and is known to be present in organisms found in biological taxa ranging from bacteria to multicellular eukaryotes. However, there have been few studies of antioxidant systems, including those that involve Prx, in the eukaryotic algae that cause HABs and induce the mass mortality of aquacultured fish. *Chattonella marina*, an especially harmful raphidophyte, is a unique phytoplankton species. Its antioxidant system is a response to high rates of ROS production and allows it to tolerate high light and high temperatures. The involvement of 2-CysPrx with the wax and wane of field blooms, its toxicity to fish, and its diurnal vertical migrations have been described in this review. Compared to other phytoplankton species, this one is particularly convenient for the study of antioxidant systems, including Prx, because of its higher production level of ROS. On the other hand, 2-CysPrx protein spots showing high expression levels, such as *C. marina*, have not yet been found in marine diatom *Thalassiosira pseudonana* [112] at a similar molecular weight and isoelectric point by analysis using 2-D electrophoresis, which suggests the relatively smaller protein expression level of 2-CysPrx in this diatom species than that in *C. marina*.

As described previously, we first found 2-CysPrx in HAB species via proteomics and determined the structure of its gene and the region of its DNA responsible for expression of that gene. A growth phase-dependent change of the level of expression of the 2-CysPrx protein was observed in a cultured strain and during a natural bloom. This pattern was also confirmed based on levels of mRNA. The clear growth phase-dependent changes in 2-CysPrx gene expression mean that this gene is a promising candidate biomarker for predicting the dynamics of *C. marina* blooms in the field.

The present review also summarized the response of 2-CysPrx gene expression to light irradiation and oxidative stress caused by H_2_O_2_. The review also reported the recent identification of five major antioxidant enzymes (Cu/Zn SOD, GPX, CAT, APX, and TRX) in *C. marina* based on RNA-seq analysis. In the analysis of gene expression using dark-acclimated *C. marina,* we observed a significant transient gene induction in Cu/Zn SOD, GPX, CAT, TRX, and 2-CysPrx during light irradiation, and this response was suppressed in DCMU-exposed cells during light irradiation. The results of this DCMU exposure test strongly support the plastid-to-nucleus retrograde signaling pathway of expression of the genes for these antioxidant enzymes. In addition, the fact that a significant increase in the expression of these genes was observed in response to treatment with more than 20 µM H_2_O_2_ suggests that the redox state of photosystem II and/or oxidative stress are triggers of expression of the six antioxidant genes. We subsequently investigated the relationship between light intensity and lipid peroxide (LPO) production in *Chattonella marina* var. *antiqua* (NIES-1, 2-CysPrx-normal-expression strain) and var. *marina* (NIES-3, 2-CysPrx-low-expression strain) subjected to supra-optimal irradiance. We found that the Fv/Fm ratio decreased and H_2_O_2_ concentrations increased when the cells of either strain were exposed to supra-optimal irradiance, and there was a significant irradiance-dependent increase in LPO in both strains. The reduction in the Fv/Fm ratio and decrease in cell density by supra-optimal irradiance were more extreme in the case of the NIES-3 strain, which contained a smaller amount of the 2-CysPrx protein than the NIES-1 strain. The implication is that the 2-CysPrx protein may contribute to the ability of the cells to tolerate supra-optimal irradiance. These results are consistent with the scenario that excessive light energy increases both the oxidative damage caused by LPO and the risk of interruption of normal cell activity (e.g., photosynthesis) and decreases the potential for phytoplankton growth. These physiological mechanisms to tolerate supra-optimal irradiance may affect the wax and wane of HABs such as *C. marina*.

In recent decades, HABs have increasingly occurred in the world’s coastal waters, where they have disrupted ecosystems, threatened public health, and adversely impacted the development of aquaculture [113]. Many studies related to the growth of HAB species have been conducted in the hope of mitigating their impacts. However, there have been few relevant studies concerned with the physiology of HAB species based on genetic information. In this review, we used proteomics and transcriptomics to explore the possible role of antioxidant enzymes in enabling *C. marina* to tolerate oxidative stress. These results provide new information about the role of antioxidant enzymes in the physiology and reproduction of this species. Moreover, our comprehensive analysis of gene expression revealed some genes closely related to the growth potential of *C. marina* that should provide information useful for the prediction of HABs in the field. However, genetic information about HAB species such as *C. marina* is meagre compared to what is known about some model organisms, because many hypothetical proteins and unknown protein genes were searched. The accumulation of more genetic information and analyses of the expression of proteins, including 2-CysPrx, will enhance understanding of the physiological mechanisms responsible for HAB formation and ichthyotoxicity.

## Figures and Tables

**Table 1:** Pearson correlation coefficients between light intensity and Fv/Fm ratio, O_2_^−^ concentration (O_2_^−^, chemiluminescence cell^−1^), H_2_O_2_ concentration (H_2_O_2_, pmol cell^−1^), H_2_O_2_ scavenging activity (CAT activity equivalence, unit cell^−1^), and LPO concentration (LPO, pmol cell^−1^). ^**^, *p* < 0.01; ^*^, *p* < 0.05.

		*Chattonella marina* var. *antiqua*	*Chattonella marina* var. *marina*
		(NIES-1)	(NIES-3)
Light intensity vs.	Fv/Fm ratio	−0.860 ^**^	−0.936 ^**^
	O_2_^−^	0.018	0.122
	H_2_O_2_	0.472 ^**^	0.540 ^**^
	CAT	−0.188	0.388 ^*^
	LPO	0.390 ^*^	0.448 ^**^

**Table 2:** Five Prx-family genes in *Chattonella marina* identified by a BlastP search using the database for stramenopiles and higher plants.

Contig (Number of Amino Acids)	Database ^a^	Classification ^b^	Retrieved Gene (Organism Name)	Accession Number of Retrieved Gene	Query Cover	E Value	Identity
659c3g1i2 (195)	S	2-CysPrx	2-cysteine peroxiredoxin, chloroplastic (*Chattonella marina* var. *antiqua*)	A0A2Z5VKM8.1	100%	5 × 10^−144^	100%
5745c0g1i1 (179)	S	PrxQ	peroxiredoxin q (*Nannochloropsis* *gaditana* CCMP526)	XP_005855889.1	93%	9 × 10^−^^69^	61%
	HP	PrxQ	peroxiredoxin Q, chloroplastic (*Erythranthe guttata*)	XP_012851266.1	84%	5 × 10^−^^52^	55%
6598c0g1i1 (221)	S	Prx4	peroxiredoxin-4, partial (*Globisporangium splendens*)	KAF1329794.1	89%	1 × 10^−^^93^	65%
	HP	2-CysPrxBAS1	2-Cys peroxiredoxin BAS1,chloroplastic (*Oryza brachyantha*)	XP_006648704.1	96%	2 × 10^−^^74^	51%
6926c0g1i1 (200)	S	Prx2	peroxiredoxin-2 (*Pythium insidiosum*)	GAY02440.1	98%	3 × 10^−^^96^	68%
	HP	Prx1	peroxiredoxin-1-like (*Rhodamnia argentea*)	XP_030535131.1	97%	9 × 10^−^^79^	61%
14890c0g1i1 (143)	S	Prx (unclassified)	peroxiredoxin (*Thraustotheca clavata*)	OQS00377.1	87%	1 × 10^−^^31^	44%
	HP	PrxIIB	peroxiredoxin-2B (*Brassica napus*)	XP_013676688.1	92%	9 × 10^−^^39^	52%

^a ^”S” means stramenopiles, and ”HP” means higher plants in the BlastP search, ^b^ Three classifications (Prx1, Prx2, and Prx4) retrieved from the database are based on mammalian criteria [82]. Other classifications are based on plant criteria [4].
